# Selecting Indicator Portfolios for Marine Species and Food Webs: A Puget Sound Case Study

**DOI:** 10.1371/journal.pone.0025248

**Published:** 2011-10-04

**Authors:** Jessi Kershner, Jameal F. Samhouri, C. Andrew James, Phillip S. Levin

**Affiliations:** 1 School of Aquatic and Fishery Sciences, University of Washington, Seattle, Washington, United States of America; 2 Northwest Fisheries Science Center, National Oceanic and Atmospheric Administration (NOAA), Seattle, Washington, United States of America; 3 Center for Urban Waters, University of Washington, Tacoma, Washington, United States of America; National Oceanic and Atmospheric Administration/National Marine Fisheries Service/Southwest Fisheries Science Center, United States of America

## Abstract

Ecosystem-based management (EBM) has emerged as a promising approach for maintaining the benefits humans want and need from the ocean, yet concrete approaches for implementing EBM remain scarce. A key challenge lies in the development of indicators that can provide useful information on ecosystem status and trends, and assess progress towards management goals. In this paper, we describe a generalized framework for the methodical and transparent selection of ecosystem indicators. We apply the framework to the second largest estuary in the United States – Puget Sound, Washington – where one of the most advanced EBM processes is currently underway. Rather than introduce a new method, this paper integrates a variety of familiar approaches into one step-by-step approach that will lead to more consistent and reliable reporting on ecosystem condition. Importantly, we demonstrate how a framework linking indicators to policy goals, as well as a clearly defined indicator evaluation and scoring process, can result in a portfolio of useful and complementary indicators based on the needs of different users (e.g., policy makers and scientists). Although the set of indicators described in this paper is specific to marine species and food webs, we provide a general approach that could be applied to any set of management objectives or ecological system.

## Introduction

Humans depend on marine ecosystems for essential goods and services, yet anthropogenic impacts frequently threaten the function and integrity of these systems [1]. There is increasing recognition that a reductionist, single-species approach to management is ineffective due to the complex interactions that characterize coupled human and natural ecosystems [2]. A promising alternative is ecosystem-based management [3], [4], which focuses on protecting ecosystem structure, function and processes to maintain ecosystem resources and services. While EBM provides a general framework for marine and coastal resource decision-making, the major challenge lies in actual implementation [5], [6].

As managers and scientists work towards implementing EBM, they require a means to track progress in achieving ecological objectives. A well-established way to track progress is through the use of indicators – quantitative measurements that serve as proxies for characterizing natural and socioeconomic systems [6]. When assembled effectively, a full suite of indicators can detect changes in ecosystem attributes and processes, providing managers with information necessary for evaluating current and past policy decisions as well as planning for the future. Despite the widespread acceptance of EBM as a strategy for managing coastal and marine ecosystems, examples of comprehensive marine EBM in practice are rare [7]. One example however, occurs in Puget Sound, Washington, USA where efforts to implement an integrated ecosystem-based management approach have been ongoing since 2007.

Puget Sound is a fjord-like estuary, covering an area of about 2,330 km^2^, including 4,000 km of shoreline. Puget Sound is part of a larger inland system situated between southern Vancouver Island and the mainland coasts of Washington State and British Columbia that encompasses the Strait of Georgia and Strait of Juan de Fuca. Puget Sound is also home to a large and increasing human population that has been, and will continue to be, an influence on the ecosystem. A growing list of threatened and endangered species, increased numbers of invasive species, significant declines in the populations of many commercially important species, degraded habitats, and hypoxic “dead zones” all point to an impaired ecosystem [8]. In response, the Puget Sound Partnership (“Partnership”) – a state-mandated effort that includes citizens, scientists, businesses, and local, state, federal and tribal governments – is working to develop and implement an ecosystem-based approach to restore, protect, and conserve Puget Sound (http://www.psp.wa.gov/). As part of the implementation process, the Partnership advocated for the development of a monitoring plan to track and assess progress towards an ecologically healthy Puget Sound [9]. A major component of this plan included the identification of environmental indicators that can capture status and trends in Puget Sound ecosystem components, as well as evaluate the effectiveness of management strategies [10], [11].

Here we report on the development of indicators for marine species and food webs in Puget Sound. Specifically, we describe an indicator evaluation process that focused on linking indicators to policy goals, evaluating indicator performance against broadly accepted scientific and social criteria, and developing indicator portfolios based on the needs of different users. In this paper we integrate a variety of familiar approaches (hierarchical frameworks, evaluation criteria, conceptual diagrams) into one step-by-step, transparent approach. Using Puget Sound as an example, we illustrate how this template can effectively be used to guide the selection of an indicator set that is scientifically credible and that resonates with policy makers. Although the set of indicators described in this paper is specific to marine species and food webs, we provide a general method for indicator evaluation that could be applied to any ecological system.

## Methods

### Hierarchical framework

**Figure 1:**
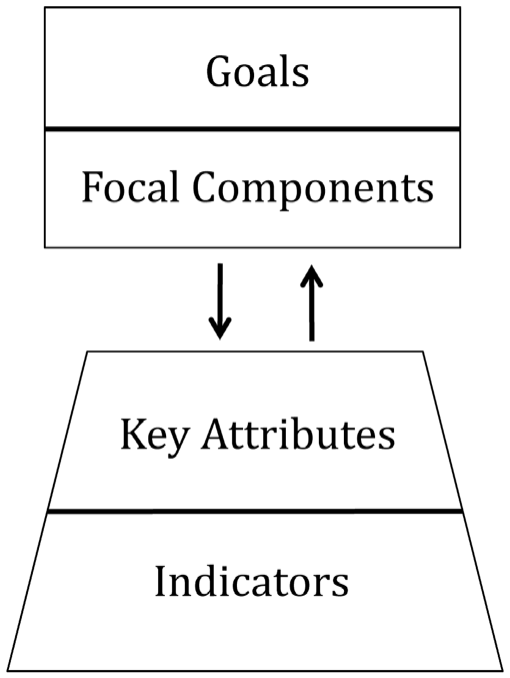
Proposed hierarchical framework for assessing and reporting on ecosystem condition in Puget Sound. *Goals* combine societal values and scientific understanding to define a desired ecosystem condition [13], [14]. *Focal components* divide a goal into its major ecological characteristics. *Key attributes* are characteristics that describe the state of a focal component. *Indicators* are metrics that reflect the structure, composition, or functioning of an ecological system and can assess changes in key attributes [14], [45]. Adapted from U.S. EPA [14].

Environmental indicators play an important role in monitoring, assessing, and understanding environmental status [12]. However, a major challenge lies in limiting the catalog of candidate indicators to a feasible subset that accurately represents the ecosystem and has the power to detect changes relevant to management goals. A straightforward approach to overcoming this challenge is to employ a hierarchical framework, which explicitly links indicators to management goals [13] (Figure 1). A hierarchical framework illustrates the progression from quantitative scientific measurements (e.g., indicators) to qualitative evaluations of whether or not societal goals are being accomplished [13]. Specifically, a well-defined framework clearly demonstrates why particular indicators were chosen and how a selected set of indicators collectively provide a balanced assessment of environmental condition and evaluate progress towards policy goals [13], [14].

We chose frameworks developed by Harwell et al. [13], the U.S. EPA [14], and Open Standards [15] as the basis for our hierarchical framework and modified them to fit the needs of the Partnership. The highest tier of the hierarchical framework, environmental *goals*, were defined by the Partnership in their Action Agenda [11]. The second tier, *focal components*, decomposes each goal into its unique ecological features. The third tier, *key attributes*, separates each focal component into its fundamental or defining characteristics. The lowest tier, ecological *indicators*, serves as a proxy for monitoring different key attributes (Figure 1; Table 1). We divided the Puget Sound ecosystem into four domains: marine, terrestrial, freshwater, and interface or ecotone, and applied the hierarchical framework within each. Here we report on developing indicators for the marine domain.

**Table 1:** Hierarchical framework applied to the selection of marine species and food web indicators for Puget Sound.

Tier	Definition	Puget Sound example
1. Goal	The broadest category of division that combines societal values and scientific understanding to define a desired ecosystem condition [13], [14].	Healthy and sustaining populations of native species, including a robust food web [11].
2. Focal Components	The major ecological characteristics of an ecosystem that can be used to organize relevant information in a limited number of discrete, but not necessarily independent categories [15].	(1) Marine Species and (2) Marine Food Webs
3. Key Attributes	The characteristics that define the structure, composition, and function of a focal component [13], [14], [15]	(1) Marine Species: *Population Size* and *Population Condition* and (2) Marine Food Webs: *Energy and Material Flows* and *Community Composition*
4. Indicators	Quantitative biological, chemical, or physical measurements that reflect the structure, composition, or functioning of an ecological system [14], [45]	(1) Marine Species: *Population Size* (e.g., harbor seal population status and trends, marine bird population estimates) and *Population Condition* (e.g., toxics in Chinook salmon, salmonid population spatial structure) and (2) Marine Food Webs: *Energy and Material Flows* (e.g., chlorophyll a) and *Community Composition* (e.g., harbor seal – food web interaction, forage fish)

Indicators listed are for example only.

#### Tier 1: goals

*Goals* are the broadest category of division that combines societal values and scientific understanding to define a desired ecosystem condition [13], [14]. Explicit descriptions of the societal values related to the condition of Puget Sound are encompassed in the statutory goals developed by the Partnership [11]. Six different goals were developed by the Partnership; however, we focus on one goal—“healthy and sustaining populations of native species in Puget Sound, including a robust food web” [11]—in the marine portion of the ecosystem to illustrate our approach. Levin et al. [16] applied this framework to several other Partnership goals including those related to habitats, water quality, and water quantity.

#### Tier 2: focal components

Focal components are fundamental characteristics of an ecosystem that provide relevant information on system structure and function [17], and are defined with regard to goals. Based on the stated goal of the Partnership, we selected two marine-specific *focal components*: marine species and marine food webs. Focal components for the remaining goals and domains are discussed in Levin et al. [16].

#### Tier 3: key attributes

Key attributes are the characteristics that define the structure, composition, and function of a focal component [13], [14], [15]. They provide a clear and direct link between indicators and focal components, and are broadly defined to allow for situations in which a single attribute can be informed by multiple indicators depending on information availability (e.g., population condition of a particular species can be tracked using data on disease for some, data on age structure for others, genetic data, etc.). Open Standards [15] recommends collecting the least amount of information that is useful to show progress, thus only a limited number of key attributes is needed. Driven by this need for simplicity and succinctness, each focal component was defined by two key attributes.

Many different attributes provide important information for understanding the status of individual species [14], [18], [19]. We selected two attributes for marine species in Puget Sound—*population size* and *population condition* (defined below). These two attributes were drawn from the literature [14], [18], [19], [20], [21], [22], [23], [24], [25], as well as a previous effort to select key attributes for Puget Sound [17].

We included the number of individuals, total biomass per unit area, and demographic rates under our definition of the *population size* attribute; similar metrics were defined by the U.S. EPA [14], Noss [20], Niemi and McDonald [19] and Fulton et al. [18]. Population abundance and biomass are key measures of the overall status of a species. More accurate assessments of species status can be obtained by monitoring demographic rates that influence changes in population size (e.g., birth and death rates, immigration and emigration). Demographic rates can also facilitate a process-based analysis of changes in population size through time.

We included organism condition, age/size structure, genetic diversity, phenotypic diversity, and spatial population structure under our definition of the *population condition* attribute. Similar metrics for population condition were described by the U.S. EPA [14], McElhany et al. [26], and by the Partnership [17]. Organism condition represents the physiological status of individuals in a population and can be used to elucidate mechanisms influencing demographic rates [14], [22]. Population age or size structure can greatly increase the predictive power of population models [27], [28]; further, changes in population size and age structure can be early signals of anthropogenic impacts [29]. The size of an organism fundamentally affects its role in an ecosystem, so understanding size structure can also help understand ecosystem dynamics [27]. Genetic diversity measures are important in assessing population condition because loss of genetic variation can reduce the productivity and viability of populations through inbreeding [30] and loss of adaptive resources [31]. There is increasing evidence that population diversity can increase both the viability of species and the services they provide to humans [32]. The spatial structure and phenotypic diversity of a population are two measures of population diversity that have been empirically linked to population productivity, reliability and viability [33].

When selecting indicators for *population size* and *population condition* key attributes, we focused on target, charismatic, vulnerable, and strongly interacting species, which represent key interests in the Puget Sound region. Target species are those fished or harvested for commercial gain or subsistence [34]. Charismatic species are those with widespread public appeal that are often used to communicate to the public about the condition of the ecosystem [34], [35]. Vulnerable species are those recognized with respect to their conservation status, for example, threatened, endangered, or of greatest conservation concern [14], [20], [34]. Strongly interacting species (e.g., keystone species, ecosystem engineers, habitat-forming species) are those whose presence, absence or rarity leads to significant changes in some feature of the ecosystem [25], [36].

Food web attributes provide important information for placing the status of individual species into a broader ecological context. We focused on two key attributes for food webs: (1) *community composition*, and (2) *energy and material flows*. These two attributes were drawn from a large literature on ecosystem structure and function [37], [38], [39], [40], [41], [42], [43].

We have adopted a broad definition of *community composition* that includes species diversity, trophic diversity, functional redundancy, and response diversity. Species diversity encompasses species richness (the number of species in the food web) and species evenness (how individuals or biomass are distributed among species within the food web [37]). Trophic diversity refers to the relative abundance or biomass of different primary producers and consumers within a food web [43]. Consumers include herbivores, carnivores or predators, omnivores, and scavengers. Functional redundancy refers to replication in the number of species that perform a single ecosystem function (i.e. nitrogen fixing), whereas response diversity describes how functionally similar species respond differently to disturbance [44]. For example, a food web containing several species of herbivores would be considered to have high functional redundancy with respect to the ecosystem function of grazing, if species of herbivores show a differential response to hypoxia, then there is also high response diversity.

The second key attribute of food webs, *energy and material flows*, includes ecological processes such as primary production and nutrient cycling, in addition to flows of organic and inorganic matter throughout a food web. Primary productivity is the capture and conversion of energy from sunlight into organic matter by autotrophs, and provides the foundation for higher trophic levels. Material flows, or the cycling of organic matter and inorganic nutrients (e.g., nitrogen, phosphorus), can mediate how energy travels through the food web.

#### Tier 4: indicators

Changes in key attributes, such as those discussed above, can be assessed through *indicators* [14], [45]. Indicators are quantitative biological, chemical, or physical measurements that reflect the structure, composition, or functioning of an ecological system [14], [45]. In an earlier effort to select indicators for Puget Sound, O'Neill et al. [46] compiled a comprehensive list of over 200 species and food web indicators, including indicators that were proposed, currently in use, or had been used in the past in Puget Sound. O'Neill et al. [46] evaluated the list of indicators using a hierarchical decision tree and framework adopted from Kurtz et al. [47] and composed a list of recommended available indicators. Based on the list of recommended available indicators in O'Neill et al. [46], as well as the addition of several new indicators, we compiled a final list of 48 potential marine species and food web indicators. Each indicator was assigned to a specific key attribute based on the literature [14], [19], [20], their previous categorization in Puget Sound [17], [48], [49], and expert opinion (Table 2).

**Table 2:** List of 48 potential marine species and food web indicators for Puget Sound.

Marine Species		Marine Food Webs	
Population Size	Population Condition	Community Composition	Energy and Material Flow
Southern Resident killer whale population trends	Toxics in harbor seals	Harbor seals – food web interaction (e.g., diet analysis)	Phytoplankton biomass
Gray whale status & trends	Smolt to adult return for wild salmonids	Benthic fish species status & trends	Chlorophyll a
Harbor porpoise/Dall's porpoise status & trends	Salmonid diversity	Bentho-pelagic fish species status & trends	
Harbor seal status & trends	Liver disease in English sole	Bottomfish species (rats & flats) status & trends	
Total run size of salmonids	Toxics in adult Chinook & coho salmon	Marine shore birds – food web interaction (e.g., diet composition)	
Marine bottomfish harvest	Toxics in Pacific herring	Forage fish status & trends	
Rockfish status & trends	Marine growth & survival of juvenile coho	Pacific herring status & trends	
Salmon & steelhead status & trends	Salmonid population spatial structure	Jellyfish abundance	
Marine resident fish species status & trends	Marine bird mortality	Shellfish (bivalve) abundance	
Marine waterfowl harvest		Macro benthic inverts abundance	
Marine bird aerial estimates (non-breeding populations)		Marine biodiversity index	
Pigeon Guillemot nesting colony trends		Marine fish & invert status & trends in marine reserves	
Marine bird status & trends during breeding season		Marine fish & invert status & trends at rocky habitats	
Marine bird breeding abundance			
Black Oystercatcher abundance			
Marine bird fishing mortality			
Glaucous wing gull abundance at nesting colonies			
Marine birds – shore-based estimates of non-breeding populations			
Western sandpiper status & trends			
Scoter & Harlequin ducks (non-breeding populations)			
Cormorant abundance at nesting colonies			
Dungeness crab abundance			
Dungeness crab harvest			
Pinto abalone status & trends			

Based on the list of recommended available indicators in O'Neill et al. [46], as well as the addition of several new indicators, a final list of 48 potential marine species and food web indicators was compiled. Each indicator was assigned to a specific key attribute (e.g., *population size*, *community composition*) based on the literature [14], [19], [20], their previous categorization in Puget Sound [17], [48], [49], and expert opinion.

### Indicator evaluation

After compiling the list of potential indicators and organizing them within the hierarchical framework (Table 2), we assembled a set of screening criteria by which to evaluate indicators and weighted criteria based on their importance to different user groups. We developed indicator portfolios by scoring candidate indicators across a range of criteria and choosing the best performers.

The methods we describe below are similar to the methods employed in a Multi-Criteria Decision Analysis (MCDA). MCDA is a tool used to determine a preference ranking among a number of available options (or in our case, indicators). Although we did not conduct a formal MCDA, we followed several of the guidelines recommended to avoid pitfalls associated with this type of approach [50], [51], [52].

#### Indicator screening criteria

A set of guidelines or screening criteria offers a consistent means to evaluate individual indicator suitability and effectiveness for monitoring programs. Evaluation criteria highlight the strengths and weaknesses of particular indicators, and allow for indicator comparison and selection within the context of specific program objectives. Table 3 lists 19 criteria that are built upon recommendations in a previous indicator report to the Partnership [46], and cover concepts from several published lists of criteria [13], [20], [53], [54], [55], [56], [57], [58], [59], [60], [61], [62].

**Table 3:** Nineteen criteria used to evaluate marine species and food web indicators for Puget Sound.

*Primary Considerations*
1) **Theoretically-sound (TS)** - Scientific, peer-reviewed findings should demonstrate that indicators act as reliable surrogates for ecosystem key attribute(s).
2) **Relevant to management concerns (RM)** - Indicators should provide information related to specific management goals and strategies.
3) **Responds predictably and is sufficiently sensitive to changes in a specific ecosystem key attribute(s) (REA)** - Indicators should respond unambiguously to variation in the ecosystem key attribute(s) they are intended to measure, in a theoretically- or empirically-expected direction.
4) **Responds predictably and is sufficiently sensitive to changes in specific management action(s) or pressure(s) (RMAP)** - Management actions or other human-induced pressures should cause detectable changes in the indicators, in a theoretically- or empirically-expected direction, and it should be possible to distinguish the effects of other factors on the response.
5) **Linkable to scientifically-defined reference points and progress targets (LT)** – It should be possible to link indicator values to quantitative or qualitative reference points and target reference points, which imply positive progress toward ecosystem goals.
*Data Considerations*
6) **Concrete (C)** - Indicators should be directly measureable.
7) **Historical data or information available (HD)** - Indicators should be supported by existing data to facilitate current status evaluation (relative to historic levels) and interpretation of future trends.
8) **Operationally simple (OS)** - The methods for sampling, measuring, processing, and analyzing the indicator data should be technically feasible.
9) **Numerical (N)** - Quantitative measurements are preferred over qualitative, categorical measurements, which in turn are preferred over expert opinions and professional judgments.
10) **Broad spatial coverage (BSC)** - Ideally, data for each indicator should be available throughout its range in Puget Sound.
11) **Continuous time series (CTS)** - Indicators should have been sampled on multiple occasions, preferably without substantial time-gaps between sampling.
12) **Spatial and temporal variation understood (STV)** - Diel, seasonal, annual, and decadal variability in the indicators should ideally be understood, as should spatial heterogeneity or patchiness in indicator values.
13) **High signal-to-noise ratio (HSN)** - It should be possible to estimate measurement and process uncertainty associated with each indicator, and to ensure that variability in indicator values does not prevent detection of significant changes.
*Other Considerations*
14) **Understood by the public and policy makers (UP)** - Indicators should be simple to interpret, easy to communicate, and public understanding should be consistent with technical definitions.
15) **History of public reporting (HR)** - Indicators already perceived by the public and policy makers as reliable and meaningful should be preferred over novel indicators.
16) **Cost-effective (CE)** - Sampling, measuring, processing, and analyzing the indicator data should make effective use of limited financial resources.
17) **Anticipatory or leading indicator (A)** - A subset of indicators should signal changes in ecosystem attributes before they occur, and ideally with sufficient lead-time to allow for a management response.
18) **Regionally/nationally/internationally compatible (CM)** - Indicators should be comparable to those used in other geographic locations, in order to contextualize ecosystem status and changes in status.
*Post-hoc Analysis*
19) **Complements existing indicators** - This criterion is applicable in the selection of a suite of indicators, performed after the evaluation of individual indicators in a post-hoc analysis. Sets of indicators should be selected to avoid redundancy, increase the complementary of the information provided, and to ensure coverage of key attributes.

*Primary considerations* provide scientifically useful, management-relevant information about the status of an indicator; *data considerations* relate to the actual measurement of an indicator, and are listed separately to highlight indicators for which data are currently unavailable; *other considerations* may be important to some user groups but are not necessarily essential for indicator performance, and are meant to incorporate non-scientific information into the indicator evaluation process.

We grouped criteria into three categories: *primary considerations, data considerations, and other considerations* [16]. *Primary considerations* are fundamental criteria that should be fulfilled by an indicator in order for it to provide scientifically sound, management-relevant information about the status of marine species and food webs. *Data considerations* relate to the actual measurement of the indicator, and are listed separately to highlight indicators that meet all or most of the primary considerations, but for which data are currently unavailable. *Other considerations* may be important to some user groups but are not necessarily essential for indicator performance, and are meant to incorporate non-scientific information into the indicator evaluation process. Advances in public policy and improvements in management outcomes, for example, may be more likely if indicators carry significant ecological information and resonate with the public [16], [63]. We applied the last criterion, “complements existing indicators”, to the final selection of the full suite of indicators in a post-hoc analysis.

#### Weighting indicator screening criteria

Although all 19 screening criteria are important to consider, it is not necessary for an indicator to meet all of the criteria to be valuable or of use for a specific application. The importance of each criterion depends on the context within which the indicators are used and the people using them. For example, Recchia and Whiteman [64] refer to the use of coarse-grained (i.e., vital signs) and fine-grained (i.e., ecosystem assessment) reporting of ecosystem status and trends. The coarse-grained level of indicator reporting is aimed at the general public and policy makers with the goal of providing a limited number of “vital signs” of the ecosystem [64]. In this instance, criteria such as cost-effectiveness, understandability, and linkable to progress targets are more important than scientifically rigorous criteria (e.g., spatial and temporal variation understood).

Conversely, the fine-grained level of indicator reporting provides a technically more robust and rigorous understanding of ecosystem structure and function, with the goal of presenting an accurate ecosystem assessment. Assessment indicators present the detailed information necessary to diagnose specific problems, develop strategies to mitigate these problems, and monitor responses of the ecosystem to management actions on multiple time scales [16]. The audience for these indicators is scientists and managers who require a detailed understanding of the ecosystem. Thus, the most important criteria include those related to the technical performance of the indicator such as theoretically-sound, responds predictably and is sensitive to changes in ecosystem key attributes, concrete, and numerical.

As regional managers and scientists consider assembling indicator sets based on user group needs, it is critical to establish the relative importance, or weight, of each criterion before evaluating indicators [60]. Rice and Rochet [60] suggest weighting criteria according to three classifications (high = essential; moderate = useful; minor = inconsequential). In addition to assigning qualitative categories to each criterion, we assigned quantitative values (i.e., essential = 1; important = 0.75; moderate = 0.5; slightly important = 0.25; negligible = 0).

In theory, *primary considerations* (e.g., theoretically-sound, relevant to management) should always be weighted highly. However, policy makers often favor indicators that resonate with the public but may not score highly for *primary considerations* (e.g., indicators related to charismatic species [65]). To incorporate this constraint on indicator selection, we developed two weighting methods: one highlighting scientific concerns (ecosystem assessment), and an alternative highlighting public considerations (vital signs) (Table 4; Table S1). The most essential ecosystem assessment criteria included: theoretically-sound, responds predictably and is sensitive to changes in ecosystem key attributes, responds predictably and is sensitive to management actions, concrete, and numerical. The most essential vital sign criteria included: relevant to management, historical data, operationally simple, numerical, continuous time series, and understood by the public and policy makers [66].

**Table 4:** Top-scoring vital sign and ecosystem assessment indicators for Puget Sound marine species and food webs.

			Population Size						Population Condition			Community Composition			E&M Flow
Criteria	VS Weight	EA Weight	Harbor seal S&T	Total run size of salmonids	Salmon & steelhead S&T	Marine bird aerial est.*	Dungeness crab harvest	Pinto abalone S&T	Toxics in adult Chinook & coho salmon	Toxics in Pacific herring	Liver disease English sole	Harbor seals –food web	Benthic fish S&T	Marine shore birds – food web	Chl a
TS	0.5	1	1	1	1	1	1	0.5	1	1	1	1	1	1	1
RM	1	0.75	0.5	1	1	1	1	1	1	1	1	0.5	1	1	0.5
REA	0.5	1	1	1	1		0.5	0.5	1	1	1	0.5	1	1	1
RMAP	0.5	1	0.5	1	1	1	0.5	1	0.5	1	1	0.5	1	0.5	1
LT	0.75	0.75	1	1	1	0	0	1	1	1	1	0	0.5	0	0.5
C	0.75	1	1	1	1	1	1	1	1	1	1	1	1	1	1
HD	1	0.5	1	1	1	1	1	1	1	1	1	0.5	0	1	1
OS	1	0.5	1	1	1	1	1	1	1	1	1	1	0.5	1	1
N	1	1	1	1	1	1	1	1	1	1	1	1	1	1	1
BSC	0.5	0.5	1	1	1	1	1	0	1	1	1	0.5	0.5	0.5	1
CTS	1	0.5	1	1	1	1	1	1	0.5	1	1	0.5	1	1	1
STV	0	0.5	1	1	1	1	0.5	0.5	1	1	1	1	0.5	1	1
HSN	0	0.5	1	1	1	1	0.5	1	1	1	1	1	0.5	1	0.5
UP	1	0	1	1	1	1	1	1	1	0.5	1	1	0	0.5	0.5
HR	0.5	0	1	1	1	1	1	1	1	0.5	1	0.5	0	0.5	1
CE	0.5	0	0.5	1	1	1	1	1	1	1	1	0.5	0	1	0.5
A	0	0.5	0			0	0	0.5				0	0	0.5	0
CM	0.25	0	1	1	1	1	1	1	1	1	1	1	0	1	1
VS Score	10.75	-	9.75	10.75	10.75	9.5	9.5	9.75	10	10	10.75	7.25	6.375	8.75	9.125
Fatal Flaw (Y/N)	-	-	No	No	No	No	No	No	No	No	No	No	Yes	No	No
EA Score	-	10	8.625	9.5	9.5	7.75	7.25	8	8.75	9.5	9.5	6.625	7.625	8.25	8.5
Fatal Flaw (Y/N)	-	-	No	No	No	n/a	No	No	No	No	No	No	No	No	No

*represents non-breeding populations.

Top scoring marine species and food web indicators were evaluated with respect to each criterion and scored. Indicators with peer-reviewed publications providing consistent and strong findings for its support received a 1; indicators with peer-reviewed documents or expert opinion providing limited support received a 0.5; and indicators with no peer-reviewed evidence, evidence against, or conflicting support received a 0. If no references were available, no score was assigned. Vital sign and ecosystem assessment scores were calculated for each indicator by multiplying each criterion score by the corresponding weight and summing across all criteria. If an indicator received a score of 0 for any essential criteria (i.e., vital sign or ecosystem assessment criteria weighted as a 1), that indicator was considered to have a fatal flaw (designated as Yes). Indicators with an empty cell for essential criteria were reported as n/a.

#### Indicator evaluation and scoring

With assistance from subject matter experts, we evaluated indicator performance against each criterion by examining peer-reviewed literature and reports (Experts included: G. Williams, NOAA; T. Good, NOAA; S. O'Neill, NOAA; T. Essington, University of Washington; I. Logan, University of Washington; S. Moore, NOAA; J. Bos, WA Dept. Ecology; K. Starke, King County Marine and Sediment Assessment Group; S. Gage, WA Biodiversity Council; and P. Dowty, WA Dept. Natural Resources). To ensure transparency and accountability for each indicator evaluation, we documented any literature used to inform the evaluation process. In addition, experts were invited to make note of key information pertaining to any or all of the criteria, which was also used to evaluate the suitability of an indicator. Extensive documentation was intended to provide sufficient evidence that an indicator met (or failed to meet) each of the specific criteria such that, based on the references and notes, an independent evaluator should be able to understand the basis for a conclusion. In addition, this approach allows for the process to be repeated and updated by others in the future. This process resulted in a matrix of cells, each containing specific references and notes summarizing indicator performance against a particular criterion (Table S2). However, in several instances available references comprised non-peer-reviewed documents and expert opinion. Consequently, where we found such documentation, we included it, while noting that it was not peer-reviewed. If references could not be found relating to a specific criterion, the cell was left blank.

Scientific support for an indicator was scored as follows: indicators with peer-reviewed publications providing consistent and strong findings for its support received a 1; indicators with peer-reviewed documents or expert opinion providing limited support received a 0.5; and indicators with no peer-reviewed evidence, evidence against, or conflicting support received a 0. If no references were available, no score was assigned. We also assigned colors to each score: 1, dark grey; 0.5, grey; and 0, light grey. The scoring matrix for all marine species and food web indicators appears in Table S1. For simplicity, our approach did not incorporate uncertainty in the scores. A number of methods exist for including uncertainty in qualitative scores such as this. For instance, one can address uncertainty by assigning a quality rating to the scores for each criterion, and use the data quality ratings to develop an overall estimate of uncertainty around each indicator ranking (e.g., [67]).

#### Final indicator scoring

Scoring acts as an initial quality control measure, culling out poor-performing indicators. Table S1 provides a quantitative summary of indicator performance against criteria, including final vital sign and ecosystem assessment scores. Final scores were calculated for each indicator by multiplying each criteria score by the corresponding weight (i.e., either vital sign or ecosystem assessment criteria weight) and summing across all criteria. Two final scores were calculated for each indicator: one vital sign and one ecosystem assessment score.

Example Equation:


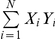
 where *X_i_* is equal to the score for criterion *i, Y_i_* is equal to the weight of the same criterion, and *N* corresponds to the number of criteria *(N* = 18). The maximum possible score for a vital sign indicator was 10.75, the maximum possible score for an ecosystem assessment indicator was 10.

#### Developing indicator portfolios

**Figure 2:**
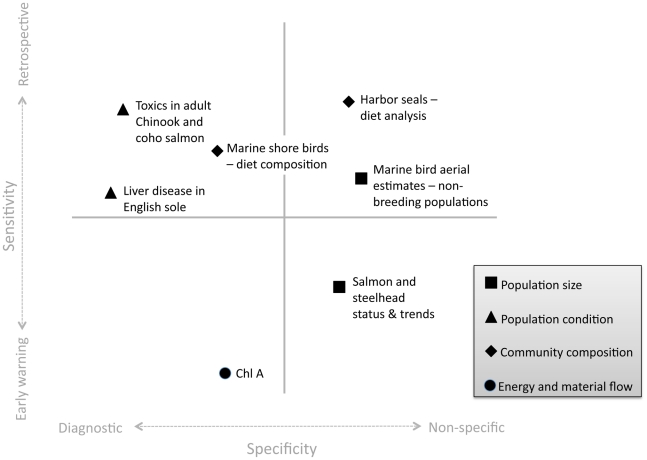
Example Vital Signs Indicator Portfolio. Top-scoring vital sign marine species and food web indicators for Puget Sound (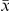 = 9.4; sd = 1.2) plotted according to whether they reliably track few (diagnostic) or many (non-specific) key attributes (x-axis) and whether they respond quickly (early warning) or slowly (retrospective) to perturbations (y-axis). A Partnership working group [66] placed each indicator in the sensitivity-specificity space. This exercise was meant to help managers heuristically think about the information conveyed by each indicator set. Figure adapted from Rapport et al. [68].

**Figure 3:**
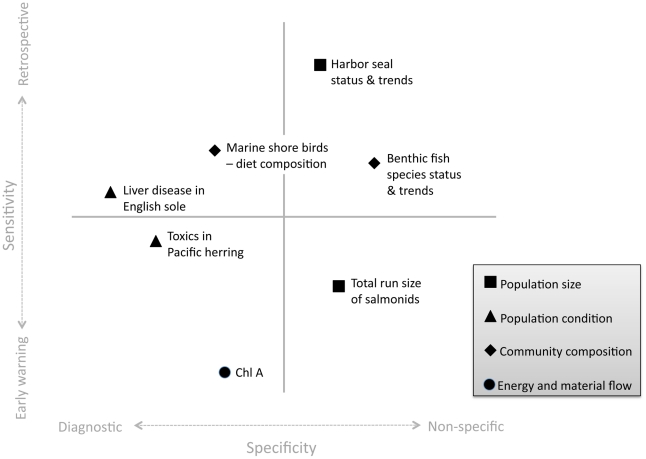
Example Ecosystem Assessment Indicator Portfolio. Top-scoring ecosystem assessment marine species and food web indicators for Puget Sound (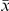 = 8.8; sd = 0.7) plotted according to whether they reliably track few (diagnostic) or many (non-specific) key attributes (x-axis) and whether they respond quickly (early warning) or slowly (retrospective) to perturbations (y-axis). Indicators categorized as diagnostic or non-specific is based on the number of attributes with which each indicator was correlated in an analysis by Samhouri et al. [70]. Similarly, indicators ranked as early warning or retrospective is based on the production to biomass ratios of each indicator species (see references in Samhouri et al. [70]). Figure adapted from Rapport et al. [68].

Scoring measures the quality of individual indicators as potential tools for managers. However, when developing a final suite of indicators it is essential not only to consider the quality of individual indicators, but also how they combine to form a well-rounded toolbox. Because managers seek to monitor ecological processes that occur at a variety of spatial and temporal scales, a functional indicator portfolio should include a diverse set of indicators that facilitate monitoring across this continuum. Rapport et al. [68] suggest selecting an indicator portfolio that fills in the 2-dimensional space represented by an axis of specificity and an axis of sensitivity (Figure 2, 3). The specificity axis describes the biological level over which an indicator integrates, where non-specific indicators can provide information about many key attributes, and diagnostic indicators provide information unique to single attributes. The sensitivity axis describes the time scale at which information about attributes are relayed. Early warning indicators provide information about impending changes in attributes before they occur, while retrospective indicators reflect changes in attributes only after they have occurred. Although retrospective indicators relay information after the fact, they can nonetheless be useful for interpreting widespread ecosystem transformation [69].

As examples, we created two indicator portfolios, one vital sign and one ecosystem assessment, for Puget Sound marine species and food webs (Figure 2, 3). Highly ranked indicators were plotted according to whether they reliably track few (diagnostic) or many (non-specific) key attributes and whether they respond quickly (early warning) or slowly (retrospective) to perturbations. For the ecosystem assessment portfolio, indicators categorized as diagnostic or non-specific is based on the number of attributes with which each indicator was correlated in an analysis by Samhouri et al. [70]. Similarly, indicators ranked as early warning or retrospective is based on the production to biomass ratios of each indicator species (see references in Samhouri et al. [70]). For the vital signs portfolio, a Partnership working group [66] placed each indicator in the sensitivity-specificity space. Importantly, this exercise was meant to help managers heuristically think about the information conveyed by each indicator set. While the vital signs portfolio did not require quantification of indicators within the sensitivity-specificity space, it may be desirable in the future.

Indicator portfolios should be kept as small as possible while still fulfilling the needs of users and relaying information at relevant time scales [60], thus each portfolio was limited to a final set of seven indicators, which included two representatives from each key attribute (except *energy and material flow*). Due to the lack of initial indicators identified for *energy and material flow*, as well as the poor performance of the other potential indicator, only one indicator was selected for both portfolios.

## Results

### Vital Signs Indicator Portfolio

The mean vital sign score for all evaluated indicators was 6.4 (sd = 3.3) out of 10.75; the mean score for the Vital Signs Indicator Portfolio was 9.4 (sd = 1.2). Our example Vital Signs Indicator Portfolio included: salmon and steelhead status and trends (10.75); marine bird aerial estimates (non-breeding populations) (9.5); toxics in adult Chinook and coho salmon (10); liver disease in English sole (10.75); harbor seals – food web interaction (e.g., diet analysis) (7.25); marine shore birds – food web interaction (e.g., diet composition) (8.75); and chlorophyll a (9.125) (Figure 2).

Table 4 provides a summary of high-scoring vital sign indicators. The highest-scoring indicators for *community composition* and *energy and material flow* were included in the vital sign portfolio by default, as was the highest-scoring indicator for *population condition* (i.e., liver disease in English sole). Two other *population condition* indicators received the same high score – toxics in Pacific herring and toxics in adult Chinook and coho salmon. We selected toxics in salmon because this indicator received higher scores than toxics in Pacific herring under *other considerations* criteria and was deemed more relevant to the public and policy makers. While two *population size* indicators received high scores (i.e., salmon and steelhead status and trends; total run size of salmonids), when plotted onto the sensitivity-specificity axis, these two indicators overlapped considerably. Both indicators tend to be diagnostic and provide specific information on population size at similar time scales. Consequently, the inclusion of both indicators in the final suite would result in redundant, rather than complementary information. We selected salmon and steelhead status and trends, and removed total run size of salmonids from the vital sign set although either indicator would have been appropriate. The next high-scoring indicators, harbor seal status and trends and pinto abalone status and trends, received the same score on their evaluations, yet both were discarded. First, harbor seal status and trends were removed to avoid redundancy with harbor seals – food web interaction, which was already selected as a *community composition* indicator. Second, abalone declines in Puget Sound likely resulted from historic overharvesting [71] making them a poor overall indicator for Puget Sound. The final *population size* indicator selected was marine bird aerial estimates (non-breeding populations). Although this indicator received the same high score as Dungeness crab harvest, it occupied a unique space on the sensitivity-specificity axis and was complementary to other indicators in the portfolio.

The vital sign portfolio and analysis was meant to be qualitative, heuristic and immediately useful for policymakers. Indeed, this approach formed the foundation of the “Dashboard Indicators” introduced by the Partnership in 2011 (http://www.psp.wa.gov/pm_dashboard.php).

### Ecosystem Assessment Indicator Portfolio

The mean ecosystem assessment score for all evaluated indicators was 5.8 (sd = 2.9) out of 10; the mean score for the Ecosystem Assessment Indicator Portfolio was 8.8 (sd = 0.7). Our example Ecosystem Assessment Indicator Portfolio included: total run size of salmonids (9.5); harbor seal status and trends (8.625); toxics in Pacific herring (9.5); liver disease in English sole (9.5); marine shore birds – food web interaction (e.g., diet composition) (8.25); benthic fish species status and trends (7.625); and chlorophyll a (8.5) (Figure 3).

Table 4 provides a summary of high-scoring ecosystem assessment indicators. Similar to the vital signs portfolio, the highest-scoring indicators for *population condition*, *community composition*, and *energy and material flow* were included by default in the ecosystem assessment portfolio. To avoid redundancy with the Vital Signs Indicator Portfolio, we selected total run size of salmonids rather than salmon and steelhead status and trends, although either indicator would have been appropriate. The next high-scoring and final *population size* indicator selected was harbor seal status and trends.

## Discussion

In any environmental management situation, key questions for decision makers include, “Are we doing the right things?” and “Have we achieved our goals?” Indicators provide the information critical to answering these questions, and, in the specific case of EBM, indicators of ecosystem structure, function, and processes can be used to assess ecosystem condition and management efficacy. Here we report on a generalized framework for selecting ecosystem indicators. While we applied the framework to marine species and food webs in Puget Sound, it can be modified to fit a diversity of ecosystems and management objectives. The flexibility of the hierarchical framework stems from its modular elements (e.g., focal components, key attributes) as well as from the ease with which the weighting of evaluation criteria can be adjusted.

The elements of the hierarchical framework are adaptable and can be adjusted to suit a diversity of management goals. For example, we have applied the framework not only to marine species and food webs, but also to additional Partnership goals related to habitats, water quality, and water quantity [16]. Further, we have applied the framework in marine, terrestrial, and freshwater ecosystems, illustrating the potential for application across a diversity of environments [16].

Criteria weights are also adaptable to any set of management objectives. For example, when considering a new monitoring program, *data considerations* criteria could receive lower weights in the interest of highlighting indicators that meet *primary considerations* (e.g., theoretically-sound, responds predictably to ecosystem attributes), but for which there are little data. In the analysis of Puget Sound marine species and food web indicators, for example, had we weighted *data considerations* less, jellyfish would likely have been selected as a good indicator of *community composition* [70].

Whereas the hierarchical framework elements and criteria weighting are adaptable, we suggest that the methods for indicator evaluations, scoring, and sensitivity-specificity plots are fundamental to this approach for several reasons. First, the methods for indicator evaluation and information scoring provided transparency and accountability when assigning indicator scores for each criterion. Oftentimes indicators are selected based solely on expert opinion, making it difficult to validate the information provided by those indicators [72]. Documenting references corroborates the basis for a conclusion and allows the scoring process to be repeated by others in the future, as new information becomes available. Additionally, allowing expert evaluators to make note of important information highlighted critical aspects of indicator performance. For example, recruiting a local expert proved vital in eliminating one indicator that otherwise performed quite well against the criteria – pinto abalone status and trends. Although the literature review supported the general use of pinto abalone as an indicator, the local expert was able to document the historic overharvesting and unlikely recovery of pinto abalone populations in Puget Sound [73].

Plotting indicators in sensitivity-specificity space can greatly increase the efficacy of indicator portfolios. In order to address management goals, a final suite of ecosystem indicators must relay the right information at the right time [68]. Plotting indicators on the sensitivity-specificity axis allows managers to tailor an indicator suite to their specific needs. In some cases, managers may want a broad portfolio that includes indicators that provide information about impending changes in marine species as well as those that reflect ecosystem-wide shifts in food webs. However, there may also be applications in which managers wish to focus on early warning, diagnostic indicators. By placing indicators in this context, even heuristically, managers can select indicators that best meet their needs.

The framework outlined in this paper presents a simple strategy for selecting a suite of ecosystem state indicators that can detect changes in ecosystem structure and function, allowing consistent and reliable reporting on ecosystem condition. It can be applied to any set of management objectives and, though the methods described here focused on indicators of ecosystem state, the approach will work for the evaluation of driver, pressure, impact, and response indicators as well (i.e., Driver-Pressure-State-Impact-Response (DPSIR) causal chain framework) [74]. Ideally, indicators should be identified for each step of the causal chain such that the full portfolio of indicators can be used to assess ecosystem condition as well as the processes and mechanisms that drive ecosystem condition. We were charged by policy makers to focus on ecosystem state indicators. However, the criteria we employed necessarily meant that the processes and mechanisms that drive ecosystem condition were captured in the *primary considerations* criteria. For example, jellyfish biomass and abundance was identified as being particularly relevant to understanding the status of forage fish in Puget Sound (i.e., increased jellyfish abundance has been associated with impairment of forage fishes) and was also linked to several pressures (*primary consideration 4*) including fishing impacts, eutrophication, habitat modification, and ballast water. Thus based on its evaluation, we understand that an increase in jellyfish biomass and abundance indicates a negative change in ecosystem condition, which is likely being driven by the pressures listed above. Although a more formal process for identifying driver, pressure, impact, and response indicators is needed, this framework provides an initial, complementary approach for completing a full situational analysis of the Puget Sound marine ecosystem.

We also recognize the need for supplementary steps in moving the indicator portfolio process forward, including the identification and evaluation of additional indicators for marine food webs, recommendations to policy makers on ways to relay this information to the public (e.g., indicator report cards), and methods for directly linking indicator values to reference levels [75]. Ultimately, however, EBM successes will depend on portfolios of indicators that scientists, managers and policy makers judge to be meaningful and useful. The approach we provide here is an important step in this direction.

## Supporting Information

Table S1 **Final scores for all 48 potential marine species and food web indicators for Puget Sound.**
(XLS)

Table S2 **Evaluations of potential marine species and food web indicators for Puget Sound.**
(XLS)
